# Prevalence, Use Behaviors, and Preferences among Users of Heated Tobacco Products: Findings from the 2018 ITC Japan Survey

**DOI:** 10.3390/ijerph16234630

**Published:** 2019-11-21

**Authors:** Edward Sutanto, Connor Miller, Danielle M. Smith, Richard J. O’Connor, Anne C. K. Quah, K. Michael Cummings, Steve Xu, Geoffrey T. Fong, Andrew Hyland, Janine Ouimet, Itsuro Yoshimi, Yumiko Mochizuki, Takahiro Tabuchi, Maciej L. Goniewicz

**Affiliations:** 1 Division of Cancer Prevention and Population Sciences, Department of Health Behaviors, Roswell Park Comprehensive Cancer Center, Buffalo, NY 14263, USA; edward.sutanto@roswellpark.org (E.S.); Connor.Miller@RoswellPark.org (C.M.); Danielle.Smith@RoswellPark.org (D.M.S.); richard.oconnor@roswellpark.org (R.J.O.); Andrew.Hyland@RoswellPark.org (A.H.); 2 Department of Psychology, University of Waterloo, Waterloo, ON N2L 3G1, Canada; ackquah@uwaterloo.ca (A.C.K.Q.); s4xu@uwaterloo.ca (S.X.); geoffrey.fong@uwaterloo.ca (G.T.F.); j2ouimet@uwaterloo.ca (J.O.); 3 Department of Psychiatry & Behavioral Sciences, Medical University of South Carolina, Charleston, SC 29425, USA; cummingk@musc.edu; 4 Ontario Institute for Cancer Research, Toronto, ON M5G 0A3, Canada; 5 Division of Tobacco Policy Research, National Cancer Center Japan, Tokyo 104-0045, Japan; iyoshimi@ncc.go.jp; 6 Japan Cancer Society, Tokyo 100-0006, Japan; mochizuki@jcancer.jp; 7 Cancer Control Center, Osaka International Cancer Institute, Osaka 537-8511, Japan; tabuchitak@gmail.com

**Keywords:** heated tobacco products, heat-not-burn, modified risk tobacco products, flavored tobacco

## Abstract

Heated tobacco products (HTPs), such as IQOS, glo, and Ploom TECH, with a variety of flavored tobacco-containing inserts, have reportedly achieved a significant market share in Japan. We analyzed data from Wave 1 of the ITC Japan Survey, a nationally representative web survey conducted in February to March 2018 among 4684 adult participants to estimate the prevalence of HTP use, describe characteristics of HTP users, and explore user preferences for HTP device and flavor. The overall prevalence of monthly HTP use was 2.7% (1.7% daily use). Virtually all HTP users were current cigarette smokers (67.8%) or former smokers (25.0%); only 1.0% of HTP users were never smokers. Among HTP users, IQOS was the most frequently reported brand used (64.5%), and menthol was the most common flavor reported (41.5%). IQOS was used more by younger respondents and those who reported daily use, while Ploom TECH was more popular among older respondents and non-daily HTP users. This is one of the first non-industry funded studies to explore the use of HTPs in Japan.

## 1. Introduction

Heated tobacco products (HTPs) are devices that heat processed tobacco rather than burn the tobacco directly in order to generate a nicotine aerosol for inhalation. Different HTP devices use different heating sources, including electric energy via a battery or carbon tip that is lit with a match or lighter. The HTP system has three common components: (1) an insert (such as stick, capsule, and pods) that contains the processed tobacco, (2) a way to heat the tobacco (i.e., battery or carbon tip), and (3) a charger for electrically heated devices [1]. While not an entirely new concept, HTPs have been around for 30 years, with the introduction of Premier by R. J. Reynolds Tobacco Company in 1988 and Accord by Philip Morris (PMI) in 1997 [2,3]. More recently, HTPs have been marketed and promoted by the cigarette companies as safer alternative to combustible cigarettes [4]. Alongside these marketing campaigns, awareness of HTPs has reached significant levels in many countries from 2015 to 2017 (e.g., 48.0% of Japanese adults [5], 38.1% of South Korean young adults [6], 19.5% of Italian adults [7], and 9.3% of adults in Great Britain had heard of HTPs [1]). In the United States (US), two studies have suggested considerable awareness of HTPs among the adult population, varying from 5.2% to 12.4% in 2016 to 2017 [8,9].

Due in part to lenient national tobacco control measures [10] and the prohibition of electronic nicotine delivery system (ENDS) marketing [11], Japan has been a testing ground for HTPs. IQOS, marketed by PMI, was first introduced in 2014, followed in 2016 by the launches of Ploom TECH by Japan Tobacco International (JTI) and glo by British American Tobacco (BAT) [11]. According to market analyst reports, Japan has the most developed HTP market of all countries worldwide, accounting for 85% of HTP sales in 2018 [12]. This status has been further cemented by the subsequent release of newer generation HTPs and flavor varieties for tobacco-containing inserts in the Japanese market (Table 1).

PMI has claimed that IQOS attained 15.5% of the overall tobacco market after just four years of its introduction [12]. While the prevalence of cigarette smoking in Japan steadily decreased over the past decade [17], the introduction of HTPs has raised concerns in achieving further tobacco control progress. HTPs might attract tobacco-naïve individuals, especially youth, with their high-tech appearance and reduced risk claims, both of which align with Japanese cultural norms around cleanliness and respect for others [18,19,20]. On the other hand, if a significant proportion of cigarette smokers switched to HTPs completely, the associated decline in smoking prevalence may confer substantial benefit towards the public’s health. HTPs promoted by the three major tobacco companies (PMI, JTI, and BAT) may employ a variety of marketing strategies to appeal to different sociodemographic user groups. Indeed, differentiated marketing strategies for cigarette smoking have disproportionately appealed to population subgroups, including youth and young adults [21,22,23], women [23], minorities [24,25], and health-concerned smokers [21].

Most of the data available on the use of HTPs in Japan has come from manufacturers and market analysts. Given the apparent popularity of HTPs in Japan, we felt it was important to independently assess the prevalence and characteristics of users. In this paper, we describe sociodemographic characteristics, product use patterns, device brand and flavor preferences among users of HTPs in Japan, including never smokers, former smokers, and current smokers (dual users).

## 2. Materials and Methods

### 2.1. Data Source

Data were from the Wave 1 International Tobacco Control (ITC) Japan Survey, a web-based survey administered by Rakuten Insight (https://insight.rakuten.com/) and collected from February to March 2018. The sampling frame of the survey was an existing Rakuten Insight panel that was nationally representative of Japanese cigarette smokers, HTP users, and non-users. Further quotas based on the region of residence, sex, and age, were applied to ensure the final sample of the survey was proportional to stratum sizes based on Japan census data. Adult residents of Japan (aged 20 (the minimum purchasing age) and older, n = 4684) were sampled as participants of this study. Participants completed an online survey, consisting of questions on HTP and cigarette use, and demographic measures, after eligibility screening. Participants were compensated with a standard Rakuten Insight incentive (points that can be redeemed as cash or merchandise) for a 35-min survey. Both survey procedures and materials were approved by the Office of Research Ethics University of Waterloo.

### 2.2. Measures

#### 2.2.1. Sociodemographic Variables

Sociodemographic variables recorded in this study were age, gender, income, and education. Age was categorized into 20–29, 30–39, 40–59, and 60 and older. Participants reported their gender as male or female. Based on Japan’s 2016 National Health and Nutrition Survey, annual household income (in Japanese Yen) was defined as follows: low (4,000,000 or less), moderate (4,000,001–6,000,000), high (more than 6,000,000), and refused/do not know [26]. Responses for education were classified as follows: low (junior high school/vocational school/high school), moderate (junior college/technical college), high (undergraduate/postgraduate), and other/refused/do not know.

#### 2.2.2. Patterns of HTP Use and Cigarette Smoking

Participants’ HTP use status was assessed by the following question: “How often, if at all, do you currently use heat-not-burn products? These include products such as IQOS, Ploom TECH, and glo”. Those who answered “daily”, “less than daily, but at least once a week”, and “less than weekly, but at least once a month” were considered **current HTP users**. Details of the survey weighting for “less than weekly, but at least once a month” HTP users are provided in Appendix A. Similarly, to assess cigarette smoking status, the following question was asked: “How often, if at all, do you currently smoke cigarettes, including both factory-made and hand-rolled cigarettes?”. Participants who responded “daily”, “less than daily, but at least once a week”, and “less than weekly, but at least once a month” were considered **current cigarette smokers**. Those who responded “Not at all; I have quit smoking completely” were considered **former smokers**; while those who responded “Not at all; I have never been a smoker” were considered **never smokers**. **Exclusive users** of HTPs or combustible cigarettes were defined as participants who only use either one of the two products and **dual users** as participants who use both products at least monthly.

Time-to-first HTP use was examined using the following questions: “How soon after waking do you usually draw your first puff on your heat-not-burn product?” for daily HTP users and “On days that you use heat-not-burn products, how soon after waking do you usually draw your first puff?” for weekly and monthly HTP users. Responses were classified into 5 min or less, 6–30 min, 31–60 min, more than 60 min, and refuse/do not know.

The number of tobacco-containing inserts per day was only assessed for daily and weekly HTP users. For daily HTP users, tobacco-containing inserts per day was assessed by the following question: “On a typical day, how many heat-not-burn tobacco sticks or capsules do you usually use?”. For weekly HTP users, we asked the following question: “On a typical week, how many heat-not-burn tobacco sticks or capsules do you usually use?” and divided the response by seven to get the number of tobacco-containing inserts per day.

#### 2.2.3. HTP Device Brand Preferences

Device brand preference was assessed using the following question: “Thinking now about the actual heating device that is used with tobacco sticks or capsules. Which of the following devices have you ever used?”. The options following the questions were “IQOS”, “glo”, “Ploom Tech”, and “other (specify)”. If participants selected more than one device brand, an additional question was asked: “Which heating device do you use most?” with the same options being given for answer.

Reason for choosing device brand was examined using the following question: “In choosing your heating device, was your choice based on any of the following?”, and the participants were instructed to respond for each of the following: (a) It may not be as bad for my health; (b) The price; (c) The taste of the sticks or capsules; (d) The design of the heating device, charging tools, etc. (Please only think about the heating/charging device, not sticks or capsules); (e) The time it takes the device to heat the sticks or capsules; (f) The advertising; (g) It is more available; (h) My friends use this product; and (i) People in the media or other public figures use this product. In each statement, the response options were “yes”, “no”, “refused”, and “do not know” thus participants could select multiple reasons for choosing a device brand.

#### 2.2.4. HTP Flavor Preferences

Flavor preference was evaluated through the following question: “What variety of flavor of tobacco sticks or capsules do you usually use?”. In addition to “refused”, “other (specify)”, “do not know”, we initially provided 22 options of tobacco sticks and capsules that were available for purchase in the Japanese market. We re-classified those flavors to tobacco, menthol, mentholated fruity, coffee, and refused/do not know. Further details of the classification of the flavor option for tobacco sticks and capsules are provided in Appendix B.

### 2.3. Statistical Analysis

We presented descriptive statistics of the study population in weighted percentage and 95% confidence interval (CI). Rao–Scott chi-square tests were conducted to both examine the difference between general characteristics of the study population and HTP use by smoking status, along with device brand preference. Normality for the number of tobacco-containing inserts per day was tested with the Shapiro–Wilk test. Due to the non-normal distribution of this variable, a Kruskal–Wallis test was employed to examine differences in the number of tobacco-containing inserts per day between each smoking status or device brand. Multiple comparisons were addressed by using Dunn’s test to produce adjusted *p*-values. Weighted multiple logistic regression analyses were performed to investigate the association between the use of each device brand and demographic characteristics, adjusting for frequency of HTP use. All statistical analyses were performed using *svy* commands in Stata SE version 14.2 (StataCorp, College Station, TX, USA). All tests were two-tailed and considered significant at *p* < 0.05. Further details on the weighting and sampling strategy are provided in the ITC Japan Survey Technical Report (https://itcproject.org/files/JP1-1.5_Technical_Report_5June2019.pdf).

## 3. Results

### 3.1. Prevalence of HTP Use and User Characteristics

Table 2 shows the characteristics of the study population stratified by smoking status. The overall prevalence of HTP use was 2.7% (95% CI: 2.4–3.0%), while the prevalence of exclusive HTP use was 0.9% (95% CI: 0.7–1.1%). Among HTP users, 67.8% (95% CI: 63.1–72.2%), 25.0% (95% CI: 20.8–29.8%), and 1.0% (95% CI: 0.4–2.0%) were current smokers, former smokers, and never smokers, respectively. A greater proportion of HTP users were male, aged 40–59, and belonged to the high-income (>6,000,000 Japanese Yen) group. The majority of HTP users who were current smokers and former smokers fell within the high education category (i.e., undergraduate or postgraduate degree).

### 3.2. Patterns of HTP Use and Cigarette Smoking

The majority of HTP users (63.4%; 95% CI: 58.9–67.6%) reported using HTPs on a daily basis, with daily use being more common among exclusive HTP users (88.3%; 95% CI: 80.5–93.2%). While the majority of HTP users among the three smoking status categories reported daily use of HTP (current smokers: 51.5%, 95% CI: 46.7–56.3%; former smokers: 86.9%, 95% CI: 77.2–92.9%; and never smokers: 100%), current smokers had the highest percentage for weekly and monthly use of HTP (19.1%, 95% CI: 16.1–22.5%; and 29.4%, 95% CI: 24.4–34.9%, respectively). HTP users most commonly reported that they use HTP 6–30 min after waking up (current HTP users: 33.8%, 95% CI: 29.7–38.1%; and exclusive HTP users: 40.9%, 95% CI: 32.2–50.1%).

The number of tobacco-containing inserts per day (median, (interquartile range)) for all HTP users and exclusive HTP users was 10.0 (2.8–15.0) and 10.0 (5.0–18.0). While the number of tobacco-containing inserts per day was not significantly different for HTP users who were former smokers and never smokers (former smokers: 10.0 (5.0–18.0) and never smokers: 15.0 (3.0–20.0)), it was significantly lower for those who also concurrently smoked cigarettes (7.0 (1.4–15.0); both *p* < 0.05).

Table 3 presents HTP use patterns and flavor preferences according to device brand preference. Although daily use was the most prevalent pattern across users of three device brands (IQOS: 69.6%, 95% CI: 63.9–74.7%; glo: 60.1%, 95% CI: 48.2–70.9%; and Ploom TECH: 49.1%, 95% CI: 39.9–58.3%), Ploom TECH users had the highest percentage of weekly and monthly HTP users compared to the other two device brands (weekly: 20.2%, 95% CI: 14.6–27.3%; monthly: 30.7%, 95% CI: 22.4–40.5%). IQOS users most frequently reported 6–30 min for time-to-first HTP use (36.6%, 95% CI: 31.3–42.3%), while glo and Ploom TECH users most often reported more than 60 min (40.3%, 95% CI: 29.6–52.0%; and 46.5%, 95% CI: 37.4–55.8%, respectively). The number of tobacco-containing inserts per day for IQOS, glo, and Ploom TECH users were 10.0 (5.0–18.0), 10.0 (5.0–18.0), and 1.0 (0.7–5.0), respectively. There was a statistically significant difference for the number of tobacco-containing inserts per day between Ploom TECH users and IQOS users, along with Ploom TECH and glo users (both *p* < 0.001).

### 3.3. Device Brand Preference

IQOS was the most prevalent device among current HTP users (64.5%, 95% CI: 60.3–68.6%), followed by Ploom TECH (21.1%, 95% CI: 17.8–24.8%), and glo (14.4%, 95% CI: 11.7–17.5%). Among exclusive HTP users, the pattern of device brand preference was similar (IQOS: 74.0%, 95% CI: 65.3–81.1%; Ploom TECH: 17.8%, 95% CI: 11.8–25.9%; and glo: 8.2%, 95% CI: 4.6–14.2%). There was a statistically significant association between device brand preference and HTP use by smoking status. IQOS was the most common HTP device brand reported by current smokers, former smokers, and never smokers (current smokers: 60.1%, 95% CI: 55.3–64.7%; former smokers: 74.8%, 95% CI: 64.1–83.2%; and never smokers: 100%).

**Figure 1:**
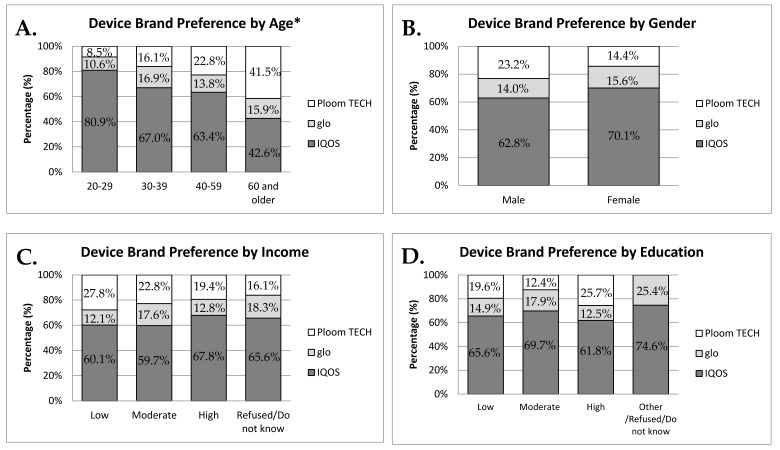
HTP device brand preference by various demographic characteristics. (**A**) Age; (**B**) gender; (**C**) annual household income; (**D**) education. The values represent a weighted percentage. * *p* < 0.001 (Rao–Scott chi-square tests).

Figure 1 shows the device brand preference by various demographic characteristics. There was an association between device brand preference and age group (Figure 1A). While IQOS was the most preferred device across all age groups, its use tended to be lower among older age groups compared to those aged 20 to 29. Conversely, Ploom TECH use was higher among older age groups, with 41.5% (95% CI: 29.9–54.2%) of those aged 60 and older using it (Figure 1A). IQOS was the most preferred device among both males and females (Figure 1B). IQOS also was the most preferred device brand across the income and education categories, while glo was the least preferred device brand (Figure 1C,D, respectively).

The association between sociodemographic characteristics and each device brand among current HTP users (adjusted for frequency of HTP use) can be seen in Table 4. Compared to those aged 20–29, those aged 30–39, 40–59, and aged 60 and older had lower odds (adjusted odds ratios (95% CI)) of using IQOS (30–39: 0.46 (0.24–0.86), 40–59: 0.36 (0.20–0.66), and aged 60 and older: 0.20 (0.09–0.43)) but higher odds of using Ploom TECH (40–59: 3.69 (1.61–8.92) and aged 60 and older: 6.32 (2.44–17.33)). No other sociodemographic characteristics were noted to be consistently associated with each device brand use. Compared to daily HTP users, those who use HTP weekly and monthly had lower odds of using IQOS (weekly: 0.61 (0.39–0.95); monthly: 0.49 (0.29–0.83)). In contrast, weekly and monthly HTP users had higher odds of using Ploom TECH (weekly: 1.89 (1.12–3.17); monthly: 2.12 (1.19–3.76)).

The most common reason for choosing IQOS was “used by friend” (68.1%, 95% CI: 62.8–73.0%), while for glo and Ploom TECH it was the “perceived reduction in health risk compared to smoking” (59.2%, 95% CI: 48.4–69.2%; and 68.4%, 95% CI: 59.2–76.4%, respectively). Among the three device brands, there were significant differences for several reasons for choosing a device brand, including price, time-to-heat, availability, used by friend, and endorsement in media (all *p* < 0.05).

### 3.4. Flavor Preference

Menthol (41.5%, 95% CI: 37.2–45.9%); tobacco (33.7%, 95% CI: 29.8–37.7%), and mentholated fruity (20.0%, 95% CI: 16.4–24.1%) were the most commonly preferred flavors among current HTP users. This flavor preference was also similar to exclusive HTP users (menthol: 47.5%, 95% CI: 38.5–56.7%; tobacco: 29.3%, 95% CI: 21.8–38.1%; and mentholated fruity: 20.3%, 95% CI: 13.8–28.9%). For HTP users who were current smokers, former smokers, and never smokers, the three most commonly preferred flavors were menthol (current smokers: 38.6%, 95% CI: 34.2–43.3%; former smokers: 49.2%, 95% CI: 38.4–60.2%; and never smokers: 65.0%, 95% CI: 28.2–89.7%), tobacco (current smokers: 35.7%, 95% CI: 31.5–40.2%; former smokers: 26.8%, 95% CI: 18.1–37.6%; and never smokers: 35.0%, 95% CI: 10.2–71.8%), and mentholated fruity (current smokers: 19.8%, 95% CI: 15.7–24.5%; and former smokers: 21.0%, 95% CI: 13.2–31.7%).

Among IQOS and glo users, the most commonly preferred flavors were menthol (IQOS: 52.6%, 95% CI: 47.0–58.2%; and glo: 51.8%, 95% CI: 41.0–62.4%), followed by tobacco (IQOS: 37.0%, 95% CI: 32.0–42.3%; and glo: 28.1%, 95% CI: 20.5–37.7%), and mentholated fruity (IQOS: 9.5%, 95% CI 5.9–15.0%; and glo: 19.0%, 95% CI: 12.4–28.0%). As there was no menthol flavor available for Ploom TECH, the most commonly preferred flavors were mentholated fruity (54.6%, 95% CI: 45.3–63.5), tobacco (29.2%, 95% CI: 21.9–37.8%), and coffee (15.0%, 95% CI: 9.5–22.9%).

## 4. Discussion

### 4.1. Prevalence and Pattern of HTP Use

This study adds to the limited body of evidence on HTPs by describing sociodemographic characteristics, product use patterns, device brand and flavor preferences among users of HTPs in Japan, including never smokers, former smokers, and current smokers (dual users). Using a national representative sample of tobacco users and validated survey instruments, we estimated that the overall prevalence of current HTP use among adults in Japan was 2.7% in 2018. To our knowledge, only two other independent studies attempted to estimate the prevalence of current HTP use in Japan. Using repeated online surveys, these studies found there was an increase in the prevalence of current HTP use (defined as use in the past 30 days) from 1.3% in 2015 to 4.7% in 2017 [5,11]. Both estimates, however, were combined percentages for current HTP use and ENDS use. While the prevalence of current ENDS use might be negligible in Japan due to the ban on the sale of ENDS, our study provides the most recent and more precise estimate of the prevalence of current HTP use. Future studies should also investigate the prevalence and pattern of HTP use among adolescents (aged 13 to 19).

Both independent and tobacco industry-funded studies have reported that the prevalence of cigarette smoking is trending downwards in Japan [17,27]. Currently, there are no data on the impact of HTP availability and use on cigarette smoking prevalence. Our data showed that the majority of HTP users are current and former cigarette smokers, with only 1.0% of HTP users who have never smoked cigarettes. Because of the limited number of participants in our survey that are HTP users who have never smoked cigarettes, we were not able to analyze this group in a detailed manner. As a HTP user is still exposed to harmful constituents [2], the uptake of HTP use among never smokers and former smokers is a crucial data point to continue assessing into the future. While the use of ENDS has previously been associated with a greater likelihood of initiating cigarette smoking among youth and young adults [28,29], it is currently unknown whether HTPs will follow a similar trajectory. Conversely, a national study in the Republic of Korea observed similar concurrent cigarette use patterns among HTP users [30]. High rates of dual use among current cigarette smokers, along with lower average tobacco inserts used per day, and longer time-to-first HTP than former or never smokers in this study suggest that HTPs may often serve as complementary products, rather than substitutes for cigarette smoking. Future longitudinal studies may show whether HTP dual use patterns are similar to those observed in other countries for ENDS, and whether dual HTP–cigarette use serve as a transitional behavioral state toward cigarette smoking cessation. Still, substantial dual use of cigarettes and HTPs warrants skepticism toward industry claims of risk-reduction.

### 4.2. Device Brand Preference

Among current HTP users, we found that IQOS was the most commonly used HTP device brand, followed by Ploom TECH and glo, consistent with prior research [11]. In addition to IQOS being introduced earlier than competitor products, PMI’s IQOS marketing strategy emphasizes important values within the Japanese culture, such as cleanliness, exclusivity, and a high-tech appearance [20], which has likely played a role in IQOS’s popularity. As new generations of HTPs are likely to come about, we plan to examine trends in device brand popularity, using the subsequent waves of data that will be collected for the ITC Japan Survey.

Among current HTP users, the odds of using IQOS were higher among young adults, while the opposite was true for Ploom TECH users, indicating a highly important sociodemographic difference between the two HTP brands. This contrasts with the PMI-funded study that reported IQOS adoption was higher in people aged 44 years and older [31]. While there have been instances where PMI’s marketing strategy for IQOS was geared towards young people [32], we did not find evidence that Ploom TECH was marketed to an older age group. Interestingly, no significant associations were observed between gender, annual household income, or education level, and use of a particular HTP device brand. IQOS users had higher odds of using HTPs on a daily basis, while Ploom TECH users had higher odds of using HTPs less frequently (weekly or monthly). Taken together, IQOS and Ploom TECH users appeared to have distinct characteristics.

While the most common reason for choosing glo and Ploom TECH was “perceived reduction in health risk compared to smoking”, it was “used by friend” for IQOS. This is a somewhat surprising finding, given that reduced risk claims are a major talking point for PMI’s IQOS marketing [33]. The reduced risk claims are primarily based on a reduction in emission of numerous toxicants from HTPs as compared to combustible cigarettes. However, a review of industry documents has shown that some substances, such as propylene glycol, glycidol, acetol, and 2-propen-1-ol, are elevated compared to the combustible cigarette, due to the higher content of humectants in the tobacco filler of the HTP inserts [34]. With IQOS being the most popular HTP device brand and its odds of use higher among young adults, the peer use aspect as a motive for using IQOS is unsurprising as peer influence has been documented to influence young adults with respect to tobacco use behavior [35]. Additionally, “endorsement in the media” as a reason for choosing device brand was significantly higher for IQOS than for glo and Ploom TECH. This corresponds with observations from a previous study in Japan, which reported an exponential increase in internet searches for IQOS immediately following the airing of a single popular television episode, in which a Japanese comedian discussed his IQOS use [11]. Future studies should aim to examine whether these differences are substantiated as the market continues to evolve, and the degree to which individual product marketing and promotional strategies may contribute to users’ reasons for adopting a particular HTP brand.

### 4.3. Flavor Preference

Regarding HTP flavor preferences, menthol was the most prevalent flavor preferred by current Japanese HTP users. Likewise, the popularity of menthol flavors in combustible cigarettes has been increasing in Japan, particularly among younger and female smokers [36]. Preference for menthol flavors among smokers has been reported to be inversely associated with age [37]. It is not surprising that HTP users, who are predominantly younger, also have preferences toward this flavor. The high popularity of menthol flavor among HTP users may have negative consequences for public health. Menthol flavor in cigarettes has been associated with increased initiation [37,38], greater nicotine dependence [39], lower quit rates [40], and higher relapse [38,39]. The tobacco industry has long utilized menthol flavor to attract users [39]. Given the wide variety of menthol flavors (and by extension other mentholated flavors, including mentholated fruity) being offered as tobacco-containing inserts, and what is known about the public health effects of menthol, the extensive use of menthol flavor for HTPs is of concern.

### 4.4. Study Limitations

There are several limitations to this study. First, data in this study were self-reported and we were not able to validate it, particularly when it comes to abstinence from cigarette smoking among exclusive HTP users. Second, this study was based on cross-sectional data; thus, causal inference should not be made. Third, even though ENDS is not commercially available in Japan, we did not evaluate the concurrent use of ENDS or ENNDS (electronic non-nicotine delivery system) among HTP users. Lastly, although the sampling and weighting strategies seek to ensure national representativeness, the possibility of excluding participants in a web-based survey cannot be ruled out.

## 5. Conclusions

In 2018, the prevalence of current HTP use in Japan was 2.7%, with most HTP users also smoking combustible cigarettes. The most popular brand device among HTP users was IQOS, followed by Ploom TECH and glo. Notable differences in age and frequency of HTP use were observed between IQOS and Ploom TECH users. Menthol was the most common flavor being used by HTP users. Given HTPs’ significant presence, tobacco regulation in Japan should also include HTPs and should be tailored to specific groups of HTP users.

## Figures and Tables

**Table 1:** Characteristics of three major heated tobacco products in Japan.

	IQOS	Glo	Ploom TECH
Device picture	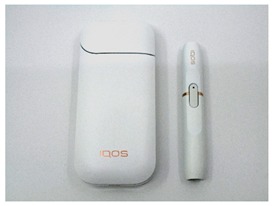	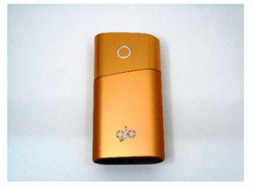	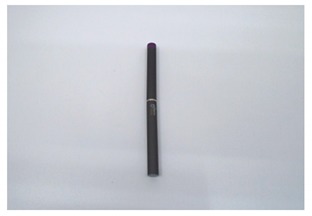
Manufacturer	Philip Morris International (PMI)	British American Tobacco (BAT)	Japan Tobacco International (JTI)
First launched	November 2014	December 2016	March 2016
Type of tobacco inserts	Stick	Stick	Capsule
Device generations	1st: IQOS 2nd: IQOS 2.4 3rd: IQOS 3 and IQOS 3 Multi	1st: glo 2nd: glo Series 2 and glo Series 2 Mini	1st: Ploom TECH 2nd: Ploom TECH+ and Ploom S *
Brand name of inserts	Marlboro Heatsticks	Kent Neostick	Mevius for Ploom TECH
Flavor variety of inserts	Balanced, Menthol, Mint, Purple Menthol, Regular, Smooth	Bright Tobacco, Citrus Fresh, Dark Fresh, Fresh Mix, Intense Fresh, Refreshing Menthol, Regular, Rich Tobacco, Smooth Fresh, Spark Fresh, Strong Menthol	Brown Aroma, Cooler Green, Cooler Purple, Red Cooler, Regular
Price ^†^	IQOS 2.4: ¥7980 [13] IQOS 3 Multi: ¥8980 [14] Marlboro Heatsticks: ¥500 [12]	glo: ¥2980 [13] glo Series 2: ¥2980 [14] glo Series 2 Mini: ¥3980 [14] Kent Neostick: ¥420 [15]	Ploom TECH: ¥2980 [14] Ploom TECH+: ¥4980 [14] Ploom S: ¥7980 [14] Mevius for Ploom TECH: ¥490 [16]

* Ploom S uses sticks instead of capsules. ^†^ For comparison, the price of combustible cigarettes is approximately ¥500 [16].

**Table 2:** Prevalence of HTP use and user characteristics.

Variable	Category	Current HTP User (n = 859)	Exclusive HTP User (n = 170)	HTP User by Smoking Status *		
				Current Smokers (n = 689)	Former Smokers (n = 115)	Never Smokers (n = 7)
		Weighted % (95% Confidence Interval)				
Prevalence		2.7 (2.4–3.0)	0.9 (0.7–1.1)	1.8 (1.6–2.1)	0.7 (0.5–0.9)	0.02 (0.01–0.06)
Demographics						
Gender	Male	76.0 (72.0–79.7)	71.8 (63.5–78.8)	78.1 (73.3–82.2)	72.8 (62.9–80.8)	62.7 (23.0–90.4)
	Female	24.0 (20.3–28.0)	28.2 (21.2–36.4)	21.9 (17.8–26.7)	27.2 (19.1–37.1)	37.3 (9.5–77.0)
				F(1.93, 1551.85) = 0.91; *p* = 0.400 ^†^		
Age (years)	20–29	16.6 (13.2–20.7)	12.9 (7.1–22.3)	18.4 (14.5–22.9)	14.8 (7.7–26.6)	-
	30–39	26.1 (22.6–29.8)	22.6 (16.2–30.5)	27.7 (23.8–32.0)	21.8 (14.4–31.4)	47.6 (15.6–81.7)
	40–59	44.0 (39.7–48.3)	55.7 (46.4–64.6)	38.4 (34.0–43.0)	54.4 (43.3–65.2)	52.4 (18.3–84.4)
	60 and older	13.3 (10.6–16.7)	8.8 (4.7–15.8)	15.5 (12.2–19.5)	9.0 (4.3–17.9)	-
				F(5.23, 4200.06) = 1.72; *p* = 0.122		
Annual Household Income	Low	15.8 (13.0–19.0)	14.3 (9.3–21.3)	16.5 (13.4–20.1)	14.9 (8.9–23.7)	11.9 (1.6–53.5)
	Moderate	22.6 (18.2–26.3)	19.3 (13.2–27.2)	24.1 (20.3–28.4)	19.6 (12.5–29.4)	10.3 (1.3–49.5)
	High	51.0 (46.6–55.3)	60.6 (51.6–68.9)	46.5 (41.8–51.2)	61.3 (50.4–71.1)	60.4 (24.0–88.0)
	Refused/Do not know	10.6 (7.9–14.1)	5.8 (3.1–10.6)	12.9 (9.4–17.6)	4.2 (1.8–9.8)	17.3 (2.4–64.1)
				F(5.42, 4351.58) = 2.18; *p* = 0.048		
Education	Low	25.9 (22.6–29.5)	24.6 (18.3–32.2)	26.5 (22.9–30.5)	21.9 (15.1–30.8)	-
	Moderate	21.4 (17.8–25.3)	22.1 (16.3–29.1)	21.0 (16.7–26.0)	18.7 (12.6–26.7)	65.0 (28.2–89.7)
	High	51.9 (47.6–56.3)	52.9 (43.9–61.7)	51.5 (46.7–56.2)	58.9 (48.3–68.6)	35.0 (10.2–71.8)
	Other/Refused/Do not know	0.8 (0.4–1.7)	0.4 (0.1–2.9)	1.0 (0.4–2.3)	0.5 (0.1–3.8)	-
				F(5.53, 4439.27) = 1.63; *p* = 0.140		
Product Use Pattern						
Frequency of HTP Use	Daily	63.4 (58.9–67.6)	88.3 (80.5–93.2)	51.5 (46.7–56.3)	86.9 (77.2–92.9)	100.0
	Weekly	16.1 (13.5–19.1)	9.9 (5.7–16.7)	19.1 (16.1–22.5)	10.8 (5.8–19.3)	-
	Monthly	20.5 (16.7–24.9)	1.8 (0.2–11.6)	29.4 (24.4–34.9)	2.3 (0.3–14.6)	-
				F(3.03, 2432.26) = 9.56; *p* < 0.001		
Time to first HTP use	5 min or less	15.4 (12.8–18.5)	17.5 (12.0–24.8)	14.4 (11.7–17.6)	15.1 (9.1–23.9)	42.0 (13.0–77.8)
	6–30 min	33.8 (29.7–38.1)	40.9 (32.2–50.1)	30.4 (62.0–35.1)	42.6 (32.1–53.7)	25.4 (3.9–74.1)
	31–60 min	15.5 (12.5–19.0)	18.9 (12.5–27.6)	13.9 (11.0–17.3)	19.8 (12.2–30.5)	10.4 (1.3–49.5)
	More than 60 min	32.8 (28.9–37.1)	20.9 (14.2–29.6)	38.5 (33.9–43.3)	20.2 (12.5–31.1)	22.2 (5.1–60.4)
	Refused/Do not know	2.5 (1.3–4.6)	1.8 (0.2–11.6)	2.8 (1.6–5.0)	2.3 (0.3–14.6)	-
				F(6.27, 5038.00) = 1.91; *p* = 0.071		
Tobacco-containing inserts per day ^‡^		10.0 (2.8–15.0)	10.0 (5.0–18.0)	7 (1.4–15.0)	10.0 (5.0–18.0)	15.0 (3.0–20.0)
Device Brand Preferences						
Device Brand	IQOS	64.5 (60.3–68.6)	74.0 (65.3–81.1)	60.1 (55.3–64.7)	74.8 (64.1–83.2)	100.0
	glo	14.4 (11.7–17.5)	8.2 (4.6–14.2)	17.2 (13.9–21.2)	8.9 (4.6–16.5)	-
	Ploom TECH	21.1 (17.8–24.8)	17.8 (11.8–25.9)	22.7 (18.9–26.9)	16.3 (9.5–26.4)	-
				F(3.68, 2921.15) = 2.69; *p* = 0.033		
Flavor Preferences						
Flavor	Tobacco	33.7 (29.8–37.7)	29.3 (21.8–38.1)	35.7 (31.5–40.2)	26.8 (18.1–37.6)	35.0 (10.2–71.8)
	Menthol	41.5 (37.2–45.9)	47.5 (38.5–56.7)	38.6 (34.2–43.3)	49.2 (38.4–60.2)	65.0 (28.2–89.7)
	Mentholated fruity ^§^	20.0 (16.4–24.1)	20.3 (13.8–28.9)	19.8 (15.7–24.5)	21.0 (13.2–31.7)	-
	Coffee	3.1 (1.9–4.9)	0.9 (0.2–3.9)	4.1 (2.5–6.7)	0.8 (0.1–5.5)	-
	Refused/Do not know	1.8 (0.9–3.4)	2.0 (0.6–6.1)	1.7 (0.7–3.7)	2.2 (0.6–7.8)	-
				F(7.47, 5995.58) = 1.16; *p* = 0.319		

* Discrepancy in total due to a small proportion of occasional smokers (less than monthly). ^†^ Rao–Scott chi-square tests accounted the complex survey design. Resulting test stats were design-based F with respective degrees of freedom for each comparison. ^‡^ Number shown are median (interquartile range). ^§^ Including mentholated vanilla.

**Table 3:** Product use pattern and flavor preference according to device brand preference.

		HTP Device Brand			*p*-Value *
		Weighted % (95% Confidence Interval)			
		IQO (n = 547)	Glo (n = 131)	Ploom TECH (n = 172)	
**Product Use Pattern**					
Frequency of HTP Use	Daily	69.6 (63.9–74.7)	60.1 (48.2–70.9)	49.1 (39.9–58.3)	0.004
	Weekly	14.8 (11.7–18.7)	16.0 (10.3–24.1)	20.2 (14.6–27.3)	
	Monthly	15.6 (11.1–21.3)	23.9 (14.0–37.5)	30.7 (22.4–40.5)	
Time to first HTP use	5 min or less	19.2 (15.4–23.6)	13.9 (8.7–21.4)	6.2 (3.4–11.2)	0.001
	6–30 min	36.6 (31.3–42.3)	26.6 (18.9–36.1)	29.6 (21.5–39.3)	
	31–60 min	15.8 (12.1–20.4)	15.2 (8.5–25.7)	16.3 (10.3–24.8)	
	More than 60 min	26.5 (21.9–31.6)	40.3 (29.6–52.0)	46.5 (37.4–55.8)	
	Refused/Do not know	1.9 (0.6–5.4)	4.0 (1.3–11.4)	1.4 (0.4–4.2)	
Tobacco-containing inserts per day ^†^		10.0 (5.0–18.0)	10.0 (5.0–15.0)	1.0 (0.7–5.0)	<0.001
**Device Brand**					
Reason for choosing specific device brand ^‡^	Perceived reduction in health risk compared to smoking	64.7 (59.2–69.8)	59.2 (48.4–69.2)	68.4 (59.2–76.4)	0.364
	Price	18.2 (14.0–23.3)	37.2 (27.8–47.6)	35.4 (26.9–44.9)	0.001
	Taste	43.3 (37.8–49.0)	36.3 (27.0–46.7)	48.4 (39.2–57.7)	0.223
	Design	37.6 (32.1–43.4)	29.4 (20.1–39.4)	39.3 (30.7–48.6)	0.768
	Time to heat	21.7 (17.8–26.2)	42.0 (31.5–53.3)	54.0 (44.6–63.1)	<0.001
	Advertising	24.9 (20.8–29.5)	23.1 (13.6–36.5)	16.9 (11.4–24.3)	0.703
	Availability	56.5 (50.7–62.1)	53.2 (42.1–64.0)	34.5 (26.4–43.6)	0.002
	Used by friends	68.1 (62.8–73.0)	41.4 (31.4–52.2)	25.8 (18.5–34.8)	<0.001
	Endorsement in media	22.9 (18.2–28.5)	8.3 (4.8–14.1)	5.9 (2.9–11.3)	<0.001
**Flavor**					
Flavor	Tobacco	37.0 (32.0–42.3)	28.1 (20.5–37.7)	29.2 (21.9–37.8)	<0.001
	Menthol	52.6 (47.0–58.2)	51.8 (41.0–62.4)	-	
	Mentholated fruity ^§^	9.5 (5.9–15.0)	19.0 (12.4–28.0)	54.6 (45.3–63.5)	
	Coffee	-	-	15.0 (9.5–22.9)	
	Refused/Do not know	0.9 (0.3–3.1)	1.1 (0.3–4.5)	1.2 (0.4–3.0)	

* Rao–Scott chi-square tests were used to account for the complex survey design. ^†^ Number shown are median (interquartile range). ^‡^ Percentage shown for those who responded yes to the statement. With other response options, added up to 100%. ^§^ Including mentholated vanilla.

**Table 4:** Multiple logistic regression examining sociodemographic characteristics by device brand preference.

		IQOS *	Glo ^†^	Ploom TECH ^‡^
		aOR (95% CI)	aOR (95% CI)	aOR (95% CI)
Gender	Male	Ref	Ref	Ref
	Female	1.14 (0.73–1.78)	0.96 (0.58–1.60)	0.84 (0.47–1.51)
Age (years)	20–29	Ref	Ref	Ref
	30–39	0.46 (0.24–0.86)	1.62 (0.78–3.35)	2.26 (0.90–5.67)
	40–59	0.36 (0.20–0.66)	1.35 (0.67–2.71)	3.79 (1.61–8.92)
	60 and older	0.20 (0.09–0.43)	1.53 (0.55–4.21)	6.52 (2.44–17.44)
Annual Household Income	Low	Ref	Ref	Ref
	Moderate	0.95 (0.51–1.76)	1.49 (0.69–3.18)	0.83 (0.39–1.75)
	High	1.49 (0.85–2.61)	1.10 (0.59–2.08)	0.55 (0.27–1.11)
	Refused/Do not know	1.22 (0.58–2.59)	1.48 (0.60–3.66)	0.56 (0.22–1.46)
Education	Low	Ref	Ref	Ref
	Moderate	1.26 (0.76–2.09)	1.16 (0.61–2.21)	0.58 (0.32–1.07)
	High	0.84 (0.54–1.31)	0.83 (0.49–1.44)	1.42 (0.83–2.45)
	Other/Refused/Do not know	1.28 (0.16–9.91)	1.66 (0.16–17.18)	- ^§^
Frequency of HTP Use	Daily	Ref	Ref	Ref
	Weekly	0.61 (0.39–0.95)	1.08 (0.61–1.93)	1.89 (1.12–3.17)
	Monthly	0.49 (0.29–0.83)	1.26 (0.62–2.56)	2.12 (1.19–3.76)

* Model 1 compares IQOS to non-IQOS device brands (glo + Ploom TECH). ^†^ Model 2 compares glo to non-glo device brands (IQOS + Ploom TECH). ^‡^ Model 3 compares Ploom TECH to non-Ploom TECH device brands (IQOS + glo). ^§^ No observation for “Other/Refused/Do not know” was recorded in Ploom TECH users.

## Appendix A. Survey Weighting for “Less than Weekly, but at Least Once a Month” HTP Users

For survey weight, the definition for current HTP users in ITC Japan Survey is daily and weekly HTP users. Meanwhile, the definition for current HTP users in this study is daily, weekly, and monthly HTP users. Monthly HTP users were, therefore, eligible for the survey in exclusive smokers and non-users categories. As a result, they were given the survey weight in accordance with each respective group. The current setting implies that for weight calculation, monthly HTP users “behave” more like HTP non-users rather than like HTP users. Similarly, monthly HTP users who were also cigarette smokers “behave” more closely to exclusive smokers than to dual users.

The additions of monthly HTP users increased variability and thus reduce power for the regression analysis. This, however, is compensated by the increase in sample size coming from including monthly HTP users and inclusion of the frequency of HTP use as one of the covariates for the regression analysis.

## Appendix B

**Table A1:** Flavor category for tobacco-containing insert products.

No	Product Name	Device Brand	Flavor Category
1	Marlboro Heatstick Balanced	IQOS	Tobacco
2	Marlboro Heatstick Menthol	IQOS	Menthol
3	Marlboro Heatstick Mint	IQOS	Menthol
4	Marlboro Heatstick Purple Menthol	IQOS	Mentholated fruity
5	Marlboro Heatstick Regular	IQOS	Tobacco
6	Marlboro Heatstick Smooth	IQOS	Tobacco
7	Kent Neostick Bright Tobacco	glo	Tobacco
8	Kent Neostick Citrus Fresh *	glo	Mentholated fruity
9	Kent Neostick Dark Fresh *	glo	Mentholated fruity
10	Kent Neostick Fresh Mix	glo	Menthol
11	Kent Neostick Intense Fresh	glo	Menthol
12	Kent Neostick Refreshing Menthol	glo	Menthol
13	Kent Neostick Regular	glo	Tobacco
14	Kent Neostick Rich Tobacco *	glo	Tobacco
15	Kent Neostick Smooth Fresh	glo	Menthol
16	Kent Neostick Spark Fresh *	glo	Mentholated fruity
17	Kent Neostick Strong Menthol	glo	Menthol
18	Mevius for Ploom TECH Brown Aroma	Ploom TECH	Coffee
19	Mevius for Ploom TECH Cooler Green	Ploom TECH	Mentholated fruity
20	Mevius for Ploom TECH Cooler Purple	Ploom TECH	Mentholated fruity
21	Mevius for Ploom TECH Red Cooler	Ploom TECH	Mentholated fruity
22	Mevius for Ploom TECH Regular	Ploom TECH	Tobacco

* Product was only available in Miyagi Prefecture, but was shown to all applicable respondents.
